# Long non-coding RNA placenta‑specific protein 2 regulates the chemosensitivity of cancer cells to cisplatin in hepatocellular carcinoma (HCC) by sponging microRNA-96 to upregulate X-linked inhibitor of apoptosis protein

**DOI:** 10.1080/21655979.2022.2056815

**Published:** 2022-04-27

**Authors:** Huixiong Wang, Xin Zhang, Xiaoting Chen, Shengbin Zhang, Zhelin Yun, Qiang Gao, Haitao Sheng, Junjie Wang

**Affiliations:** a Department of Hepatobiliary Surgery, Hospital of Inner Mongolia Baotou Steel, Baotou, Inner Mongolia, China; b Department of Pathology, The Second People’s Hospital of Foshan Affiliated Foshan Hospital of Southern Medical University, Foshan, Guangdong, China; c Department of Plastic surgery, Hospital of Inner Mongolia Baotou Steel, Baotou City, Inner Mongolia, China

**Keywords:** Hepatocellular carcinoma, PLAC2, XiaP, cisplatin, chemosensitivity

## Abstract

This study was conducted to investigate the roles of lncRNA PLAC2 and XiaP in hepatocellular carcinoma (HCC). HCC and paired non-tumor tissues were collected from 62 HCC patients who received cisplatin-based treatment. At 0, 2, and 4 months of post-cisplatin-based therapy, blood samples (5 ml) were collected from all patients and prepared plasma samples. LncRNA PLAC2 expression in tissue and plasma samples was determined by RT-qPCR. The interactions between lncRNA PLAC2 and XiaP in HCC cell lines were assessed by overexpression experiments. Cell viability and apoptosis under cisplatin treatment were analyzed by MTT assay and cell apoptosis assay, respectively. The direct interaction between lncRNA PLAC2 and miR-96, which can target XiaP, was analyzed by performing RNA–RNA pulldown assay. It was observed that lncRNA PLAC2 was upregulated in HCC tissues than in non-tumor tissues. LncRNA PLAC2 expression in HCC tissues was not affected by HBV and HCV but upregulated after cisplatin-based treatment. Similarly, cisplatin treatment of HCC cells increased PLAC2 expression. LncRNA PLAC2 and XiaP overexpression increased viability and decreased apoptosis of cisplatin-treated HCC cells, while lncRNA PLAC2 knockdown decreased viability and increased apoptosis of cisplatin-treated HCC cells. Western blot analysis showed that lncRNA PLAC2 increased XiaP protein accumulation, while lncRNA PLAC2 siRNA silencing decreased XiaP expression in HCC cells. LncRNA PLAC2 and miR-96 directly interacted with each other, while they failed to regulate the expression of each other. In conclusion, lncRNA PLAC2 negatively regulates the chemosensitivity of HCC cells to cisplatin, possibly by sponging miR-96 to upregulate miR-96.

## Graphical abstract

**Figure:**
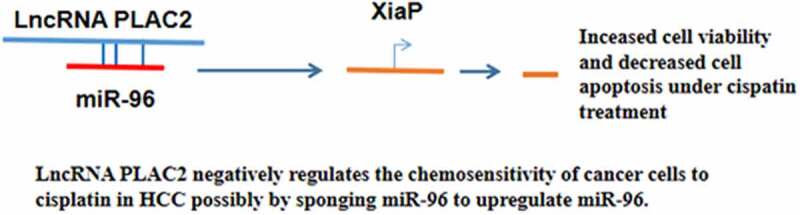

## Highlights

- PLAC2 was upregulated in HCC tissues and after cisplatin-based treatment.
- PLAC2 and XiaP overexpression increased cell viability, while PLAC2 knockdown decreased viability under cisplatin treatment.
- PLAC2 increased XiaP while PLAC2 decreased XiaP at protein level in HCC cells.
- PLAC2 and miR-96 directly interacted with each other but failed to regulate the expression of each other.

## Introduction

Hepatocellular carcinoma (HCC) is considered one of the most diagnosed malignancies and a major cause of cancer-related deaths [1]. It has been reported that HCC is responsible for more than 660,000 deaths every year worldwide [2]. Early diagnosis of HCC is technically challengeable [3]. Patients with HCC are prone to develop macrovascular invasion, intrahepatic metastasis, and even distant tumor metastasis [4]. However, there is no cure for metastatic HCC [5]. Chemical drugs, such as cisplatin, are frequently used for the treatment of HCC patients at advanced stages [6]. However, drug resistance is frequently observed, leading to poor long-term outcomes [7].

The inhibition of drug resistance is critical for the survival of advanced cancer patients. More and more genetic factors, such as multidrug resistance-associated proteins, have been demonstrated to be involved in the development of chemoresistance [7]. X-linked inhibitor of apoptosis protein (XiaP) is known as an apoptosis protein 3 inhibitor [8]. XiaP protects cells from apoptosis, thereby contributing to cancer development [9]. It has been reported that XiaP is involved in chemoresistance, and XiaP inhibition provides a potential target for novel cancer therapies [10]. STAT1 is also a key player in cancer cell apoptosis and has crosstalk with XiaP [11]. A recent study identified lncRNA PLAC2 as a novel tumor suppressor in glioma, possibly mediated by STAT1 [12]. Moreover, it is predicted that lncRNA PLAC2 may interact with miR-96, which can directly target XiaP [13]. Therefore, lncRNA PLAC2 may indirectly interact with XiaP via STAT1 or miR-96. Therefore, this study was conducted to investigate the involvement of lncRNA PLAC2 and XiaP in HCC.

## Materials and methods

### Study subjects

The present study included 62 HCC patients selected from 156 HCC patients who were admitted at the Hospital of Inner Mongolia Baotou Steel from October 2016 to October 2018 after the Hospital Ethics Committee approved this study, and informed consent was obtained. Ethics approval document is presented in Supplemental file 1. The inclusion criteria were as follows: (1) new HCC cases, (2) stage IV patients not appropriate for surgical resection but received cisplatin-based treatment, and (3) expected survival time >4 months. The exclusion criteria were as follows: (1) patients with recurrent HCC (previously diagnosed and treated), (2) therapies were initiated, and (3) multiple clinical disorders were diagnosed. HBV and HCV infections were detected by performing sensitive PCR. Among the 62 patients, 20 cases were HBV positive, 14 cases were HCV positive, 15 cases were both HBV and HCV positive, and 13 cases were both HBV and HCV negative. All patients were educated with the details of the principle of this study.

### HCC tissues, plasma, and cell lines

Liver biopsy was performed for the diagnosis. During the biopsy, HCC tissues and paired non-tumor tissues (0.013–0.18 g) were collected. All tissue specimens were confirmed by histopathological examinations. Blood (5 ml) was collected from each patient in EDTA tubes at 0, 2, and 4 months of post-cisplatin-based therapies (65 mg/m^2^ on the 1st, 15th, and 29th days in a 1-month treatment course) and centrifuged at 1,200 g for 12 min to prepare plasma samples. Two HCC cell lines, SNU-475 and SNU-387 (ATCC, USA), were cultured in RPMI-1640 media (Cat# 11,875,093, Thermo Fisher, Shanghai, China) containing 100 IU/ml penicillin–streptomycin solution (Cat# 15140122, Thermo Fisher, Shanghai, China).

### Transfections

pcDNA3-PLAC2 and pcDNA3-XiaP vectors, NC siRNA, lncRNA PLAC2 siRNA, miR-96 mimic, and NC miRNA were obtained from GenePharma (Shanghai, China). SNU-475 and SNU-387 cells were harvested at 70–80% confluence, and 12 nM lncRNA PLAC2 or XiaP expression vector, empty pcDNA3 vector (negative control, NC), 50 nM lncRNA PLAC2 siRNA, or negative control siRNA (negative control, NC) was transfected using Lipofectamine 2000 (Cat# 11668019, Invitrogen, Shanghai, China).

### RT-qPCR

HCC and normal tissues were mixed with RNAzol reagent (Cat # R4533, Sigma-Aldrich, USA) to extract total RNAs. To investigate the effects of cisplatin on PLAC2 expression, SNU-475 and SNU-387 cells were first treated with cisplatin at doses of 0, 1, 2, and 4 μg/ml for 24 h before RNA extraction. To determine mature miRNA expression, poly(A) tail was added to RNA samples using Poly(A) Tailing Kit (Cat# AM1350, Thermo Fisher, Shanghai, China). Precision nanoScript2 Reverse kit (Cat# RT-NanoScript2, Primerdesign Ltd., UK) was used for cDNA preparation. Accumulation of lncRNA PLAC2 and XiaP was analyzed using RT-qPCR with GAPDH as the internal control and normalized using the 2^−ΔΔCt^ method [14]. Primer sequences were 5'-TGTGGCCCAAACTCAGGGATACA-3' and 5'-AGATGACAGTGGCTGGAGTTGTCA-3' for lncRNA PLAC2, 5'-GCACCGTCAAGGCTGAGAAC-3' and 5'-ATGGTGGTGAAGACGCCAGT-3' for GAPDH; 5'-GCGGTGCTTTAGTTGTCAT-3' and 5'-CGGGTATATGGTGTCTGATA-3' for XiaP, and 5'-TTTGGCACTAGCACATTTTTG-3' and oligo d(T) for miR-96.

### Western blot

Total proteins were extracted from SNU-475 or SNU-387 cells (10^5^) in 1 ml RIPA solution (Cat# 89900, Thermo Fisher, Shanghai, China). Denatured samples were separated on 10% SDS–PAGE gel and transferred onto PVDF membranes. The membranes were blocked at room temperature for 2 h in 5% nonfat milk and incubated first with rabbit polyclonal primary antibodies against GAPDH (1:900, ab181602, Abcam, Cambridge, UK) and XiaP (1:900, ab233164, Abcam, Cambridge, UK) at 4°C overnight and then with HRP-labeled secondary antibody of (1:1,000, goat anti-rabbit, Cat # MBS435036, MyBioSource, Shanghai, China). The signals were developed with ECL™ Detection Reagent (Cat# GERPN2105, Sigma-Aldrich, Shanghai, China) and photographed using myECL™ Imager (Thermo Fisher, Shanghai, China).

### MTT assay

MTT assay was performed as previously reported [15]. In brief, 3 × 10^4^ SNU-475 or SNU-387 cells were resuspended in 1 ml RPMI-1640 media, placed in a 96-well plate with 0.1 ml per well, and incubated with 0, 2, or 4 μg/ml cisplatin (Cat# 15663–27-1, Sigma-Aldrich, Shanghai, China) with three replicates for each dosage at 37°C for 24 h in a humidified incubator with 5% CO_2_. After that, cells in each well were incubated with 10 µl MTT (Cat# M2003, Sigma-Aldrich, Shanghai, China) for 4 h, and OD values at 450 nm were measured.

### RNA–RNA pulldown assay

NC and lncRNA PLAC2 transcripts were prepared using MEGAscript™T7 Transcription Kit (Cat # AM1334, Thermo Fisher Scientific, Shanghai, China) and labeled with biotin using the Pierce™ RNA 3’ End Biotinylation Kit (Cat # 20163, Thermo Fisher Scientific, Shanghai, China). The obtained Bio-NC and Bio-lncRNA PLAC2 were transfected into cells. Cell lysates were prepared at 48 h after transfection and subjected to RNA pulldown using Dynabeads (Thermo Fisher Scientific, Shanghai, China). The accumulated miR-96 was determined using RT-qPCR.

### Cell apoptosis assay

Cells were cultured in media containing 4 μg/ml cisplatin for 48 h. After that, cells were collected, stained with FITC and PI using Annexin V-FITC Apoptosis Detection Kit (Cat# APOAF-20TST, Sigma-Aldrich, Shanghai, China), and sorted using flow cytometry. Data were analyzed using BD FACSuite™ software version 1.0 (BD Biosciences, CA, USA).

### Statistical analysis

All data were analyzed using GraphPad Prism 6 software. Differences between two groups and among multiple groups were compared using unpaired *t*-test and ANOVA Tukey’s test, respectively. The 62 HCC patients were divided into high and low lncRNA PLAC2 level groups, and associations between patients’ clinical data and lncRNA PLAC2 expression in HCC were analyzed with chi-squared test. *p* <0.05 was statistically significant.

## Results

### LncRNA PLAC2 was upregulated in HCC but not affected by HBV and HCV infections

**Figure 1.**
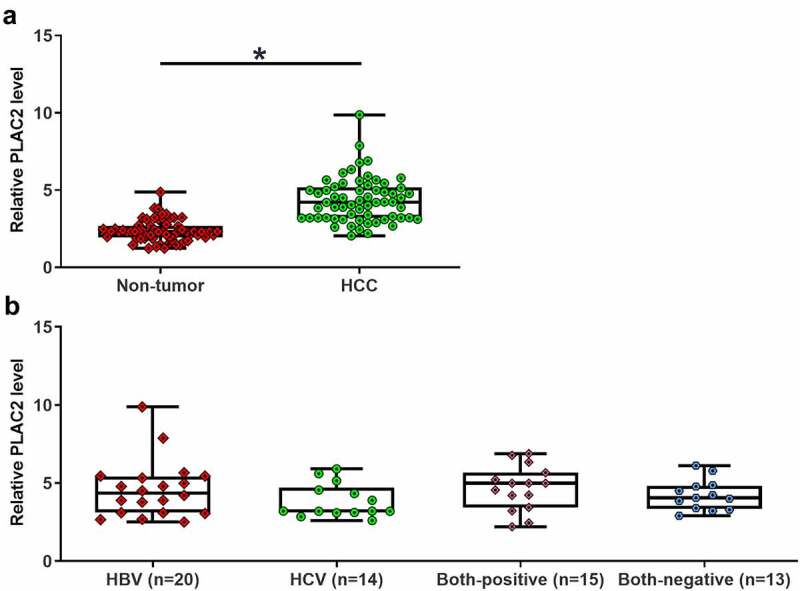
LncRNA PLAC2 accumulation in HCC. RT-qPCR results showed that lncRNA PLAC2 levels are increased in HCC tissues (*p < 0.05) (a) but not affected by HCV and HBV (b).

To study the differential expression of PLAC2 in HCC, paired HCC and non-tumor tissue samples were collected from 62 HCC patients. The 62 patients included 36 males and 26 females. The 62 HCC patients included 20 HBV-positive cases, 14 HCV-positive cases, 15 double-positive cases, and 13 double-negative cases. LncRNA PLAC2 expression levels in HCC and non-tumor tissues from these 62 patients were detected by RT-qPCR. It was observed that lncRNA PLAC2 level was 1.73-fold higher in HCC tissues than in non-tumor tissues (Figure 1(a), *p* < 0.05). LncRNA PLAC2 levels were not affected by HCV and HBV (Figure 1(b)). Moreover, lncRNA PLAC2 in HCC was not closely correlated with patients’ age, gender, tumor size, tumor number, and tumor differentiation stages (Table 1). Therefore, lncRNA PLAC2 upregulation is involved in HCC.

**Table 1.** Associations between patients’ clinical features and lncRNA PLAC2 expression in HCC tissues

Parameters	Group	Total	PLAC2		Chi-squared	*p*
			Low	High		
Sex	Male	36	20	16	1.06	0.30
	Female	26	11	15		
Age	<50	30	14	16	0.26	0.61
	≥50	32	17	15		
Tumor size	<5	26	11	15	1.06	0.30
	≥5	36	20	16		
Tumor number	Solitary	43	20	23	0.68	0.41
	Multiple	19	11	8		
Differentiation stage	High	21	8	13	1.80	0.41
	Moderate	23	13	10		
	Low	18	10	8

### LncRNA PLAC2 was upregulated by cisplatin

**Figure 2.**
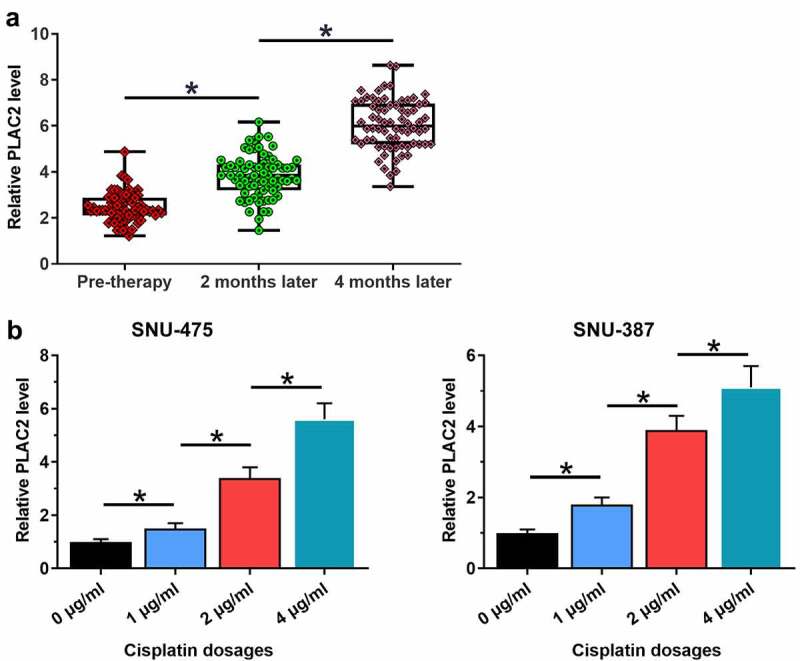
LncRNA PLAC2 was upregulated by cisplatin. Plasma lncRNA PLAC2 levels at 0, 2, and 4 months after cisplatin treatment were measured using RT-qPCR and compared using ANOVA. LncRNA PLAC2 expression levels were significantly increased with prolonged cisplatin treatment (a). SNU-475 or SNU-387 cells were treated with 0, 1, 2, and 4 μg/ml cisplatin for 24 h. Cisplatin upregulated lncRNA PLAC2 expression (*p < 0.05) (b).

Development of chemoresistance is common in the treatment of HCC. To this end, the potential involvement of lncRNA PLAC2 in cisplatin-induced chemoresistance in HCC patients was analyzed. Plasma lncRNA PLAC2 levels at 0, 2, and 4 months of post-cisplatin therapies were measured using RT-qPCR. It showed that plasma lncRNA PLAC2 levels increased significantly with the prolonged treatment (Figure 2(a), *p* < 0.05), showing a 1.90-fold increase at 2 months of post-therapy compared to that at pre-treatment and a further 1.51-fold increase at 4 months compared to that of 2 months. SNU-475 or SNU-387 cells were treated with cisplatin at doses of 0, 1, 2, and 4 μg/ml for 24 h. Cisplatin upregulated lncRNA PLAC2 expression in a dose-dependent manner (Figure 2(b), *p* < 0.05), showing a fivefold increase at 4 μg/ml in both cell lines. Therefore, lncRNA PLAC2 may participate in cisplatin-induced chemoresistance in HCC.

### LncRNA PLAC2 positively regulated cell viability under cisplatin treatment

**Figure 3.**
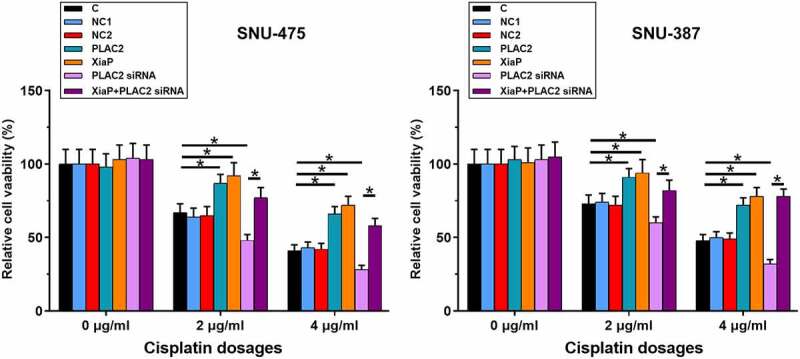
LncRNA PLAC2 negatively regulated cell viability under cisplatin treatment. Cell apoptotic data showed that compared to NC and C two controls, lncRNA PLAC2 and XiaP overexpression increased, while lncRNA PLAC2 silencing decreased HCC cell viability under the treatment of 2 and 4 μg/ml cisplatin. In addition, XiaP overexpression significantly reduced the effects of lncRNA PLAC2 silencing on cell viability (*p < 0.05).

Cell viability reflects the responses of cancer cells to chemotherapy. Therefore, the role of lncRNA PLAC2 in regulating HCC cell viability under cisplatin treatment was analyzed. Cells were transfected with pcDNA3-PLAC2 or pcDNA3-XiaP expression vector or lncRNA PLAC2 siRNA. At 24 h post-transfections, overexpression of lncRNA PLAC2 and XiaP and silencing of lncRNA PLAC2 were achieved (Supplemental Figure S2). After that, cells were treated with 0, 2, and 4 μg/ml cisplatin for another 24 h, followed by MTT assay to measure cell viability. LncRNA PLAC2 and XiaP overexpression increased HCC cell viability, while lncRNA PLAC2 l silencing decreased HCC cell viability under 2 and 4 μg/ml cisplatin treatment (Figure 3, *p* < 0.05). XiaP significantly reduced the effect of lncRNA PLAC2 silencing on cell viability (*p* < 0.05). However, lncRNA PLAC2 and XiaP showed no significant effect on HCC cell viability at 0 μg/ml cisplatin. The roles of lncRNA PLAC2 and XiaP in regulating HCC cell apoptosis under 4 μg/ml cisplatin treatment (48 h) were studied using cell apoptosis assay. It was observed that lncRNA PLAC2 and XiaP decreased, while lncRNA PLAC2 silencing increased HCC cell apoptosis. XiaP significantly reduced the effect of lncRNA PLAC2 silencing on cell apoptosis (Supplemental Figure S1, *p* < 0.01). Therefore, PLAC2 and XiaP may increase the viability and decrease the apoptosis of HCC cells under cisplatin treatment.

### LncRNA PLAC2 positively regulated the expression of XiaP in HCC cells

**Figure 4.**
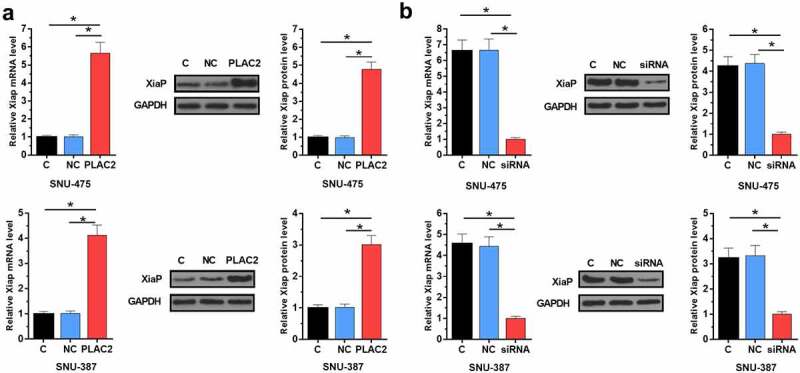
LncRNA PLAC2 positively regulated XiaP expression in HCC cells. Compared to NC and C groups, lncRNA PLAC2 overexpression upregulated (a) while lncRNA PLAC2 silencing downregulated (b) XiaP in HCC cells (*p < 0.05).

To study whether XiaP is the downstream effector of PLAC2 in regulating HCC cell proliferation, the role of PLAC2 in regulating XiaP expression was analyzed using Western blot and RT-qPCR. We found that PLAC2 significantly increased XiaP expression level in HCC cells at both mRNA (more than fourfold) and protein (more than threefold) levels (Figure 4(a), *p* < 0.05). LncRNA PLAC2 silencing deceased XiaP expression level in HCC cells at both mRNA (more than fourfold) and protein (more than threefold) levels (Figure 4(b), *p* < 0.05). Therefore, PLAC2 can positively regulate XiaP in HCC cells. Original Western blot images were presented in Supplemental file 2.

### LncRNA PLAC2 can sponge miR-96 in HCC cells

**Figure 5.**
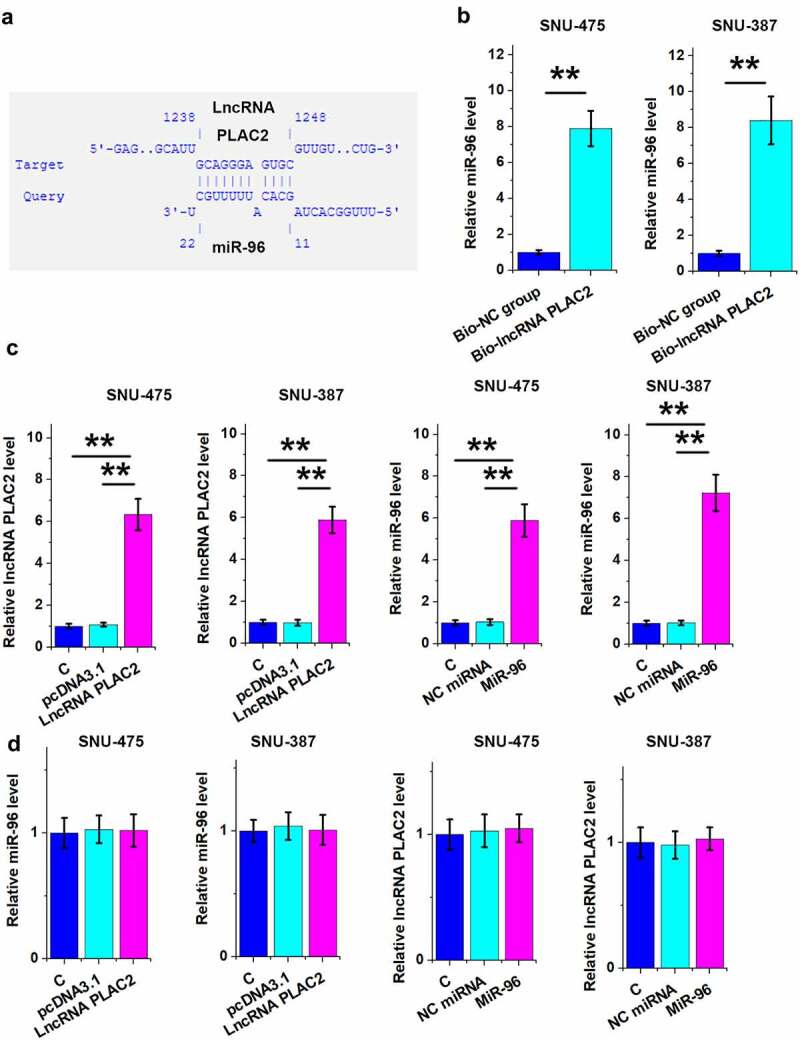
LncRNA PLAC2 sponged miR-96 in HCC cells.

To explore the mechanism mediating lncRNA PLAC2-regulated XiaP expression, the direct binding of lncRNA PLAC2 to miR-96, which can directly target XiaP [13], was predicted using IntaRNA 2.0. It was shown that miR-96 might bind to lncRNA PLAC2 (Figure 5(a)). Their direct interaction was analyzed using RNA–RNA pull-down assay. Compared to Bio-NC group, miR-96 was increasingly accumulated in Bio-lncRNA PLAC2 pulldown complex, suggesting that they have a direct interaction (Figure 5(b), *p* < 0.01). To further explore their relationship, lncRNA PLAC2 and miR-96 were overexpressed (Figure 5(c), *p* < 0.01), and the role of lncRNA PLAC2 and miR-96 in regulating the expression of each other was analyzed using RT-qPCR. Interestingly, they failed to affect the expression of each other (Figure 5(d)). Therefore, lncRNA PLAC2 is unlikely a target of miR-96 but may sponge miR-96.

The direct binding of PLAC2 to miR-96 was predicted using IntaRNA 2.0 (a). The direct interaction between PLAC2 and miR-96 was analyzed using RNA–RNA pull-down assay (b). To explore the relationship between PLAC2 and miR-96, lncRNA PLAC2 and miR-96 were overexpressed in HCC cells (c). The role of lncRNA PLAC2 and miR-96 in regulating the expression of each other was analyzed using RT-qPCR (d) (**p < 0.01).

## Discussion

This research studied the role of lncRNA PLAC2 in HCC. We found that lncRNA PLAC2 was upregulated in HCC and further upregulated by cisplatin. In addition, lncRNA PLAC2 regulated the sensitivity of HCC cells to cisplatin via interacting with XiaP.

Studies have shown that one lncRNA can exert similar functions in different types of cancer. For example, lncRNA HOTAIR promotes tumor metastasis in almost all types of cancers by reprogramming chromatin state [16]. LncRNA MALAT1 enhances tumor metastasis in most but not all types of cancers [17]. LncRNA TUG1 promotes glioma cancer cell apoptosis [18,19]. However, TUG1 regulates miR-138-5p to promote cervical cancer progression [16]. It is known that lncRNA PLAC2 inhibits cancer progression by interacting with STAT1 and RPL36 [12]. In this study, we observed upregulated lncRNA PLAC2 expression in HCC tissues. In addition, lncRNA PLAC2 positively regulates HCC cell viability under cisplatin treatment. Therefore, lncRNA PLAC2 is likely an oncogene in HCC. However, more experiments and clinical trials are needed to test this possibility. In addition, it is possible that lncRNA PLAC2 exerts opposite functions in different types of cancers. The role of lncRNA PLAC2 in other types of cancers cannot be speculated based on its known roles in different cancer types.

XiaP can be regulated by certain lncRNAs in cancer biology [20]. For instance, XiaP-AS1 interacts with Sp1 to promote XiaP transcription [20]. In this study, we observed that lncRNA PLAC2 is upregulated by cisplatin in both HCC tissues and cell lines. It is known that lncRNA PLAC2 can regulate STAT1 activity [12], and STAT1 can regulate cancer cell viability by regulating the translation of XiaP and Bcl-xl [21]. We found that lncRNA PLAC2 positively regulated XiaP expression to affect the sensitivity of HCC cells to cisplatin. Moreover, lncRNA PLAC2 was found to sponge miR-96, which can directly target XiaP [13]. Therefore, lncRNA PLAC2 may sponge miR-96 to upregulate XiaP. We speculated that the interaction between XiaP and lncRNA PLAC2 may also be mediated by STAT3. We will test this possibility in the future.

With the increased understanding of lncRNAs in HCC progression and the development of chemoresistance in HCC cells [22–24], novel approaches are expected to be developed to increase the efficiency of chemotherapy. Our study is limited by the small sample size. Our findings should be further verified with larger sample size. Moreover, the interaction among XiaP, miR-96, and lncRNA PLAC2 characterized in the present study should also be assessed by *in vivo* animal model experiments.

## Conclusions

LncRNA PLAC2 is upregulated in HCC and can be further upregulated in both patients and HCC cell lines by cisplatin. Moreover, the expression of PLAC2 is not correlated with HBV and HCV infections. Under cisplatin treatment, PLAC2 promotes the survival of HCC cells possibly through the upregulation of XiaP by serving as an endogenous competing RNA for miR-96. Overall, this study characterized a novel PLAC2/miR-96/XiaP pathway involved in the development of chemoresistance of HCC cells to cisplatin. This pathway participates in HCC through HBV- and HCV-independent pathways. This pathway may be targeted to improve the sensitivity of HCC cells to chemotherapy.

## Supplementary Material

Supplemental Material

## Data Availability

We accept reasonable requests.
