# Supplementary figures and images for: Long non-coding RNA placenta‑specific protein 2 regulates the chemosensitivity of cancer cells to cisplatin in hepatocellular carcinoma (HCC) by sponging microRNA-96 to upregulate X-linked inhibitor of apoptosis protein
Source: Bioengineered. 2022 Apr 27;13(4):10765–73. doi: 10.1080/21655979.2022.2056815 (PMC9208526; doi:10.1080/21655979.2022.2056815)

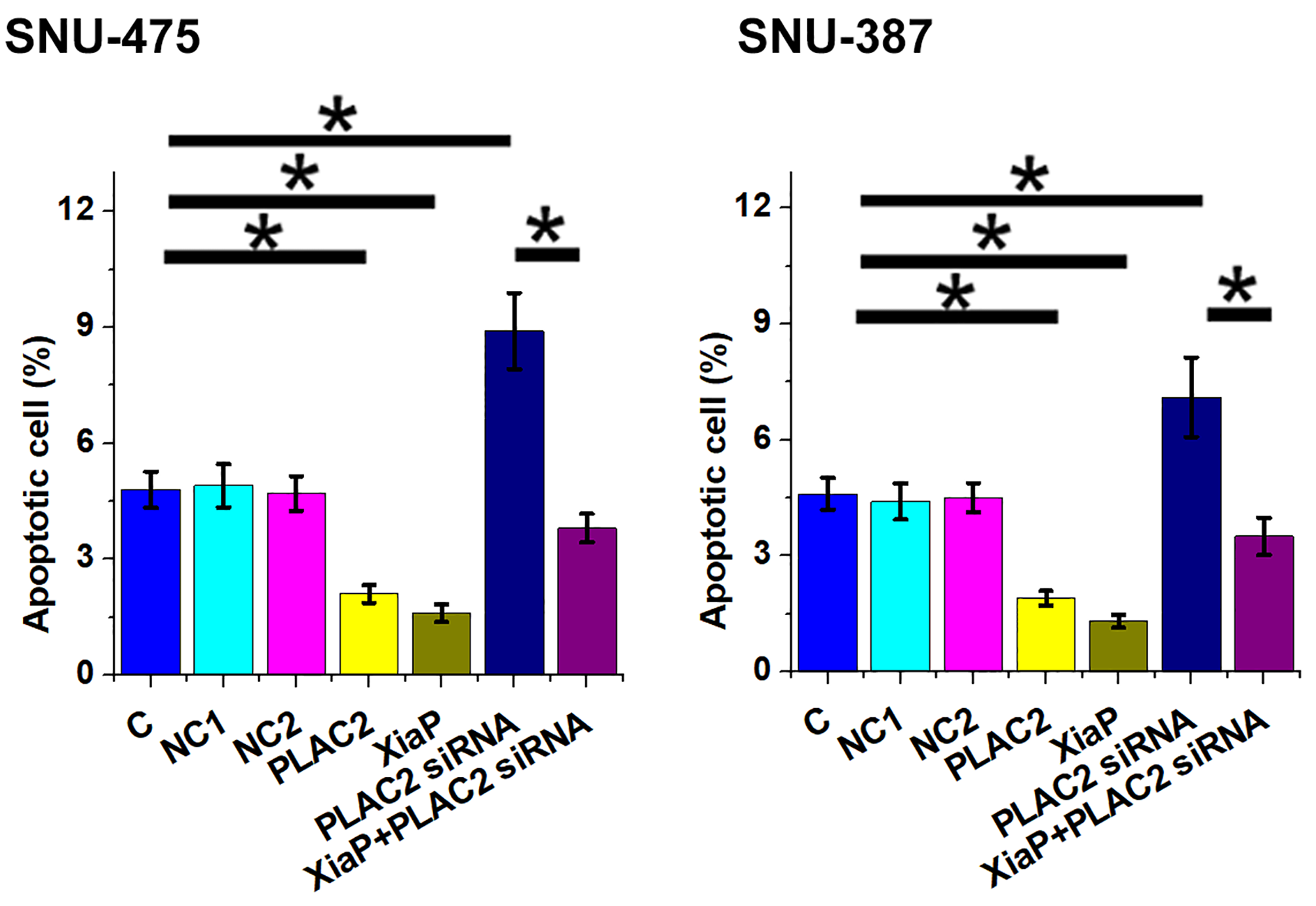

Supplement: Supplemental Material [file KBIE_A_2056815_SM9622.zip › supplementary/Supplemental Fig1.tif]

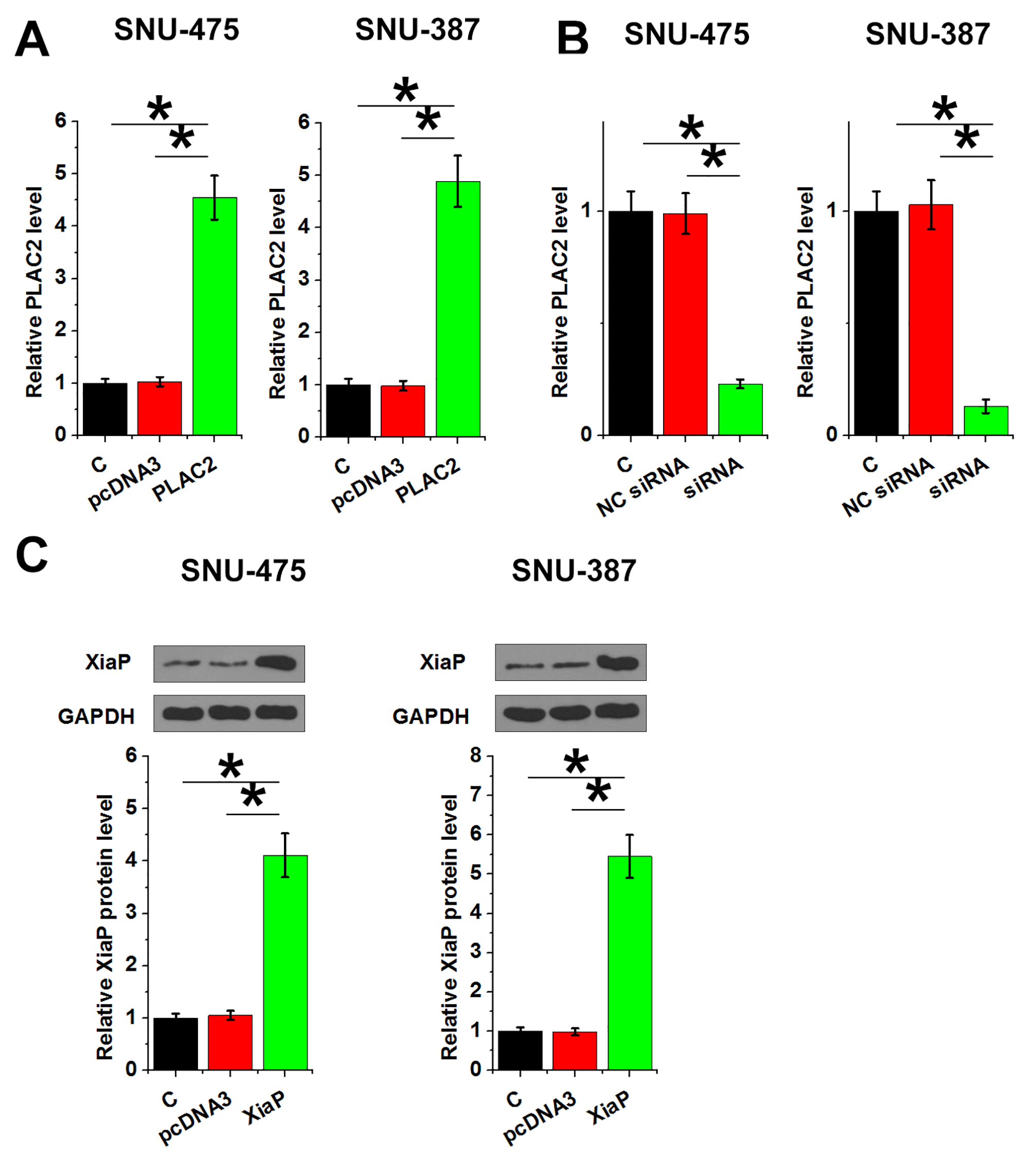

Supplement: Supplemental Material [file KBIE_A_2056815_SM9622.zip › supplementary/Supplemental Fig2.tif]
